# Intuition and Deliberation in the Stag Hunt Game

**DOI:** 10.1038/s41598-019-50556-8

**Published:** 2019-10-16

**Authors:** Marianna Belloc, Ennio Bilancini, Leonardo Boncinelli, Simone D’Alessandro

**Affiliations:** 1 Sapienza University of Rome, Rome, Italy; 2 IMT School of Advanced Studies, Piazza S.Francesco 19, 55100 Lucca, Italy; 3 University of Florence, Florence, Italy; 4 University of Pisa, Pisa, Italy

**Keywords:** Social behaviour, Human behaviour

## Abstract

We present an incentivized laboratory experiment where a random sample of individuals playing a series of stag hunt games are forced to make their choices under time constraints, while the rest of the players have no time limits to decide. We find that individuals under the time pressure treatment are more likely to play *stag* (vs. *hare*) than individuals in the control group: under time constraints 62.85% of players are *stag*-hunters as opposed to 52.32% when no time limits are imposed. These results offer the first experimental evidence on the role of intuition and deliberation in strategic situations that entail social coordination. In interpreting our findings, we provide a discussion on ruling social conventions in daily-life interactions.

## Introduction

The recent literature in judgment and decision-making has shown an upsurge of interest to understand prosociality from a dual process perspective^1^. Dual process theories of decision-making^2^ are well established in cognitive and social psychology. They suggest that humans make decisions under two modes of reasoning, namely *intuition*, fast and relying on heuristics, and *deliberation*, slow and based on careful scrutiny of costs and benefits^3–5^.

 Following this insights, a number of recent contributions have run incetivized experiments to investigate the effects that the mode of reasoning has on prosocial behavior in a variety of games^6,7^, such as: prisoner dilemmas and public good games^8,9^, dictator games^9–11^, ultimatum games^12,13^, deception games^14^, and allocation decisions^15^.

In this paper we consider the stag hunt game, which can be interpreted as a social dilemma involving prosociality^16^. In the basic game, the opposition between coordination on *stag* and coordination on *hare* can be seen as a parable for social situations in which coordination can be pursued on two different levels: coordinating on better rewarding, but necessarily collaborative actions, and coordinating on less rewarding actions, which do not require collaboration. Accordingly, coordinated play on either action can be interpreted as a social norm, i.e., a social convention^17,18^.

Even if the stag hunt game has been widely investigated with experimental methods^19,20^, to our knowledge, there is no previous attempt to empirically assess the effects that the tension between intuition and deliberation has on the choice between *stag* and *hare*. Some experimental evidence is available for pure coordination games^21^, suggesting that intuition leads to rely more on culturally focal options.

In this work, we manipulate the mode of reasoning by imposing a 10-second time constraint on decision-making^6,22^. While response times are related to the mode of reasoning in important respects^23^, the interpretation of results obtained under time pressure requires careful consideration^9,24^.

As already noted^25^, different theories of dual process cognition identify different attributes to, respectively, intuition and deliberation^5,26^. A large body of this literature relates intuition to automatic and unconscious processes that occur extremely fast, possibly in less than a second^27^; several contributions on the relationship between prosocial behavior and ego depletion^10,13,28–30^ or cognitive load^12,31^ take this perspective. Other contributions define intuition as a mode of reasoning that is not fully unconscious and automatic, but entails some reflection in the form of heuristics^32–34^; in these studies, intuition is assumed to be substantially slower than in the previous approach. Different definitions of intuition can, at least in part, explain opposing results in the analysis of intuitive behavior and prosociality^1,35,36^. Indeed, while some researchers contend that intuition induces cooperative behaviors while reflection stimulates selfishness^8,33,37,38^, others argue that deliberation and reflection act as a hurdle to selfish impulses and lead to prosociality and cooperation^13,39,40^. Finally, some studies find no effect of the adoption of intuition on cooperation^41,42^.

The interpretation of intuition as a mode of reasoning well fits our experimental setting where a 10-second time constraint is used to inhibit reliance on deliberation. According to this interpretation, the distinction between intuition and deliberation recalls the distinction between instinctive and contemplative decision-making, implying that both processes involve conscious reasoning and require a minimum amount of time and reflection^43^. In this perspective, intuition may well lead to the adoption of heuristics that need some reflection to be applied, as is typical of strategic interactions. In the last section of the paper, we explore the implications of our results under the Social Heuristics Hypothesis^8^, an approach that is recently gaining attention as a conceptual framework to identify the heuristics that underlie intuitive decision-making, when interpreted as a mode of reasoning.

## Methods

We conducted an experiment at the CESARE Laboratory of LUISS - Guido Carli University of Rome, programmed in z-Tree^44^. The participants were recruited from a pool of students at LUISS - Guido Carli University using ORSEE^45^ with the only restriction of a more or less equal gender balance. The overall number of participants in the experiment was 185, divided in eight sessions.

The participants were all asked to play series of four different one-shot two-player stag hunt games (the same games, but in different order) with a perfect stranger matching protocol (i.e., two subjects never interact more than once) and no feedback information. All the individuals faced the same experimental setting (same laboratory, same instructions, same instructions reader), with the only exception of the treatment (time pressure vs. control, as we explain below).

**Figure 1:**
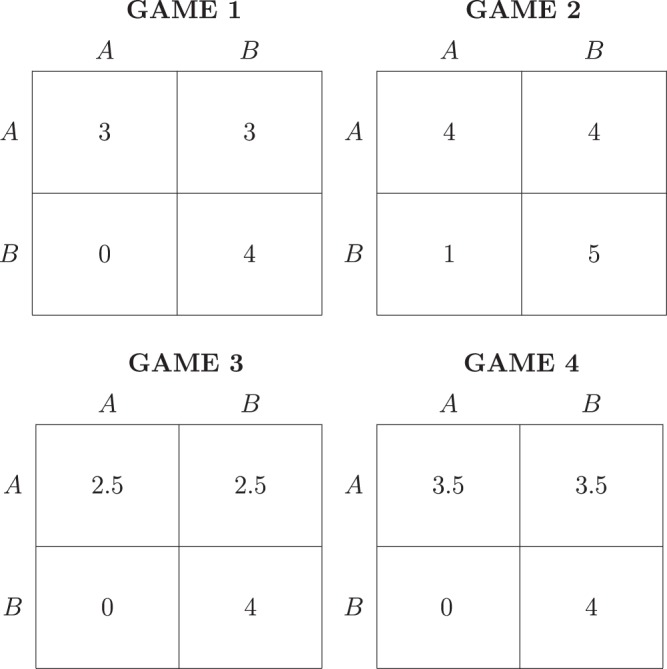
The four stag hunt games of the experiment.

Denoting *hare* by *A* and *stag* by *B*, Fig. 1 reports the payoff earned by a generic player (the game is symmetric) in the four games, depending on own choice (row) and opponent’s choice (column): e.g., in Game 1 a payoff of 0 is earned if *B* is played against *A*. The games differ by their prominence of coordinating on *stag*, as captured by the size of its basin of attraction: i.e., 1 minus the minimum probability of the opponent playing *stag* which makes playing *stag* a best response. Game 1 and Game 2 have the same basin of attraction of *stag* (Game 2 is the transformation of Game 1 where one point is added to each outcome): playing *stag* is best reply if and only if the probability of the opponent playing *stag* lies in the interval $[3/4,1]$, which gives a basin of attraction of *stag* equal to 1/4. Game 3 has the largest basin of attraction of *stag*, equal to 3/8, while Game 4 has the smallest, equal to 1/8.

In order to make the conversion of the payoffs in monetary units easier for the participants, we set an exchange ratio payoff/euro of 1:1. For instance, a realized payoff of four gave the right to be paid four euro.

Individuals were randomly assigned to one of two different treatments. The first (control group) had no time constraint on decision-making. Four sessions of this treatment were run, for a total of 97 participants, 51 men and 46 women. The second (time pressure treatment group), by contrast, was asked to take an action under a time limitation of 10 seconds in each game. Four sessions of this treatment were also run, for a total of 88 participants, 47 men and 41 women.

In all the sessions, Game 1 was the first to be played, while Game 2, 3, and 4 where played in different order in the various rounds (see Table B.1 of the SI Appendix, for descriptive details of each session). The control group and the time pressure treatment group were balanced under relevant respects such as gender, family background, age, education, and experience in game theory and their applications (see Table B.2 of the SI Appendix).

All decisions were made individually and there was no interaction among the participants in the experiment (except for the determination of the payoffs that took place at the end of the experimental session). Players were not allowed to use any electronic device or to write on paper. Since the simultaneous start of each of the four games for all the participants would have led some of them to wait until all the others were ready to start the new game (possibly altering the effect of the time pressure treatment depending on how quickly an individual played previous games), we opted to let players make their choices independently of the timing of the opponent’s choice. No feedback information was provided to the participants during play. At the end of the session, when all the players had made their decisions, pairs were formed in each game and payoffs computed.

After playing the four games, the participants were asked to fill a series of questionnaires in order to collect information regarding their individual characteristics, aspects of their life, and their usual modes of reasoning. In particular, questionnaires were about: general questions (such as family background, education, etc), Rational-Experiential Inventory (40 items), Cognitive Reflection Test, risk love, and trustfulness (see Table B.3 of the SI Appendix for details).

After all the questionnaires were answered, payoffs were communicated and the participants received their payments. They were paid an amount of euro equal to the game payoff plus two euro of show up fee. Average total payoff was 11.24 (the average payoff per game was 2.81) for a session that lasted around 45 minutes. Informed consent was obtained by all the players. In particular, the participants were informed that data would be used anonymously for scientific purpose only. The experiment was conducted in accordance with regulations and relevant guidelines for experiments with human subjects of the CESARE lab at LUISS and therefore approved by the LUISS’ ethics committee.

## Data Analysis

### Descriptive evidence

**Figure 2:**
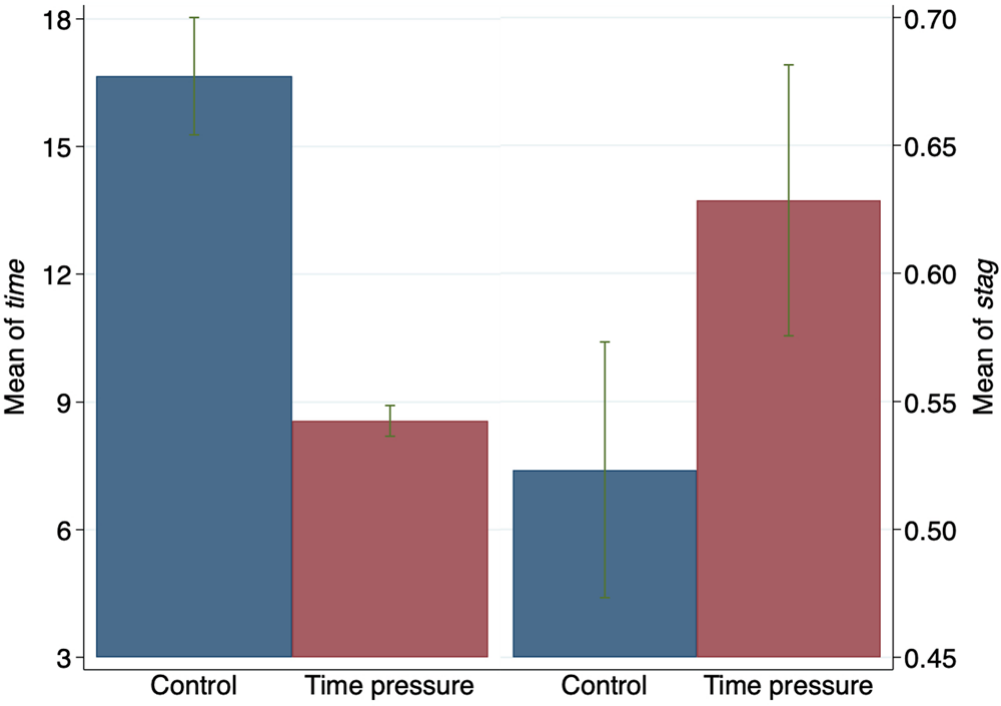
Average time in seconds for decision (left) and average play of *stag* (right), in control and time pressure treatments (confidence intervals at 95%). In both cases the two-sample *t*-test for mean equality rejects the null hypothesis at least at the 5% level of statistical significance.

The time pressure treatment was aimed at forcing individuals to respond more quickly when playing the games than they would do in the absence of the time constraint. Figure 2, left panel, shows that the treatment effectively reduced average response times. The time lapse spent by participants to make a decision varied remarkably between groups (the same holds when we consider games one-by-one). As displayed in the figure, the overall average time spent to make a decision is 16.65 seconds under the control and 8.55 seconds under the time pressure treatment. The two-sample mean comparison *t*-test proves that the difference between these two numbers is statistically significant at the 1% level^a^^1^. This result also holds when we compute the average time for each game separately considered.

Figure 2, right panel, reports the fractions of individuals choosing *stag*, also computed by treatment group. It reveals that the fraction of *stag*-hunters in the control group is equal to 52.32%, while that in the time pressure group is 62.85%, with the difference being statistically significant at the 5% level (again according to the two-sample mean comparison *t*-test). This is a first piece of evidence documenting that the participants facing the time constraint chose *stag* more often than those in the control group.

### Regression analysis

To identify the effect of the time pressure treatment on the play of *stag*, we run OLS regressions. In estimation, the number of observations drops from 740 (185 × 4) to 711 because, under the time pressure treatment, 29 choices were not made within the 10-second constraint and were discarded, as a consequence, from the estimation sample (see Section B.4 of the SI Appendix for a robustness check that our conclusions are not driven by excluding these data points). Results are shown in Table 1, where we report both heteroskedasticity robust standard errors (round brackets) as well as standard errors clustered at the individual level (square brackets)^b^^2^.

**Table 1:** Main results - OLS Regressions.

Variable	(1)	(2)	(3)	(4)
*pressure*	0.1053	0.0996	0.1301	0.1289
	(0.0370)	(0.0364)	(0.0369)	(0.0365)
	[0.0525]	[0.0505]	[0.0507]	[0.0505]
*risk love*			0.0976	0.0950
			(0.0379)	(0.0374)
			[0.0522]	[0.0520]
*basin*				1.0764
				(0.2014)
				[0.1669]
R-squared	0.011	0.067	0.106	0.140
Day Fe	No	Yes	Yes	Yes
Session Fe	No	Yes	Yes	Yes
Round Fe	No	Yes	Yes	Yes
Individual controls	No	No	Yes	Yes

The number of observations is 711. The dependent variable is *stag*_*it*_ = 1 if individual *i* chose *stag* in round *t* and =0 otherwise. Individual controls include: gender, post-graduate education, father’s education, mother’s education, CRT scores, REI40 measures, inexperience in game theory and lab experiments, measure of trust in others (see Section B.3 of the SI Appendix for variables’ definitions). Standard errors in round brackets are heteroskedasticity robust, while those in square brackets are clustered at the individual level.

Column (1) reports regression output when the only independent variable is a dummy equal to one if the individual subject was under time pressure treatment and to zero otherwise (fixed effects not included). Here, the impact of the time constraint on the choice of *stag* is positive and statistically significant at any conventional level. This implies that being under time pressure increases the probability of playing *stag* by about 10 percentage points. Such positive effect is confirmed when we also include the three sets of fixed effects, day, session, and round fixed effects (column (2)) and, in addition, a number of individual controls (column (3)). Among the individual controls, only risk love has a systematic statistically significant effect on individual choices, suggesting that less risk averse individuals are more likely to choose *stag* (estimated coefficients on the other variables are, thus, not reported to save on space; see Section B.3 of the SI Appendix for details).

Column (4) lists estimates when we further include in the regression a variable which is equal to the size of the basin of attraction of *stag* in the various games and which is meant to capture the impact of the game relative payoffs. While the size and statistical significance of the time pressure coefficient remain similar to those reported in the previous columns, the coefficient on the basin of attraction turns out positive and statistically significant at any conventional level. This suggests that the larger the basin of attraction of *stag*, the larger the set of beliefs that justifies the choice of *stag* and, as a consequence, the more likely that an individual takes this action.

## Discussion

A prominent conceptual framework that looks at the relationships between the mode of reasoning and prosocial behavior is the so-called Social Heuristics Hypothesis (SHH, hereafter)^8,32^. According to the SHH, intuition relies on heuristics shaped by daily-life experience, which enable individuals to make decisions quickly. In particular, the SHH predicts that intuition favors the adoption of the strategies that have resulted, on average, to be most advantageous in daily-life social interactions, i.e., that maximized average payoff over a sufficiently long period of interaction. By contrast, deliberation would occur when individuals resist the impulse to rely on social heuristics and reflect more deeply upon the situation at hand, choosing the payoff maximizing strategy case-by-case. So, deliberation is typically slower than intuition.

Most experimental studies on the SHH have focused on one specific social dilemma, the prisoner dilemma (or the public good game), where *defection* is a strictly dominant action for selfish individuals^6^. In this context, the SHH predicts that, if *cooperation* pays more than *defection* in daily-life prisoner dilemmas, then individuals relying on intuition cooperate more than those relying on deliberation, even in one-shot games. In particular, intuitive behavior can foster *cooperation* in the lab because, by relying on such mode of reasoning, individuals fail to recognize that the game they are playing is actually one-shot^46^.

Some social interactions fit the stag hunt game by their own nature. Others resemble a stag hunt game, although they apparently have different nature^16^. In these cases, the problem of social coordination arises in an extended setting because of some additional characteristics of the game (e.g., reputation effects) or the type of interaction involved (e.g., repeated interaction) that, once taken into account, generate a reduced-form game of the stag hunt type. As an example, consider a prisoner dilemma where, besides the standard Nash equilibrium in which *defection* is played, also the act of *cooperation* by all the players can be enforceable as an equilibrium if players care about reputation effects or fear to be sanctioned by the other players (or by third-party actors and institutions).

Which kind of behavior is induced by the adoption of the different modes of reasoning in strategic situations that resemble the stag hunt game? The SHH suggests that individuals relying on intuition tend to choose the action corresponding to the ruling norm in the social environment where they typically interact with others (the social environment they face in everyday life), whereas individuals relying on deliberation opt for the payoff maximizing action in the situation at hand from time to time. The fact that promoting intuition vs. deliberation seems to have no effect on cooperative behavior if individuals live in non-cooperative environments^15^ is consistent with this idea.

These theoretical predictions, however, cannot help interpret behavior in stag hunt games without further assumptions. Indeed, stag hunt games have no dominant strategy (such as *defection* in the prisoner dilemma). In the type of games considered here, two Nash equilibria exist, so that behavior by rational agents (which approximates individual decision-making under deliberation) remains substantially indeterminate. Also, the literature that employs evolutionary game theory^47,48^ to study the long-run selection between *stag* and *hare* is inconclusive. The long-run coordination occurs on *hare* when interaction is totally random^49^ or totally unconstrained^17,47^. Coordination on *stag* is, instead, selected when interactions are reasonably constrained in number and not fully random^50^ or when individuals have also to choose a location where to take an action between *stag* and *hare*^51–53^. Finally, evolutionary arguments suggest that payoff-dominant outcomes at the population level are likely to be selected when group competition is at work. Indeed, in human history, reproduction and struggle for existence have made collaboration among individuals extremely effective^54–56^. A mixed outcome can also emerge in the presence of strong homophily motives^57^.

Pulling these reflections together, our experimental evidence can be interpreted as consistent with the idea that, under the assumption that the SHH is at work, *stag* is the ruling social norm in real life stag hunt interactions.

Since *stag* can be interpreted as a more collaborative and trusting behavior than *hare*, our contribution can be placed in the recent stream of literature investigating under what circumstances the choice between intuition and deliberation leads to prosociality^58^. Yet, even if our results support the idea that intuition is more conducive to prosociality than deliberation, we cannot exclude that opposing effects of fast decision-making could be found when behavior is driven by automatic processes^13^ or that deliberation could promote, instead, moral (e.g., Kantian) reasoning^59^.

To better qualify the kind of prosociality involved in the stag hunt game and to facilitate comparison with the existing literature, we offer an interpretation in the light of the distinction between pure cooperation (i.e., cooperation in settings where cooperating is not in one’s own interest) and strategic cooperation (i.e., cooperation in settings where cooperating can maximize individual payoffs)^6^. If we interpret the choice of *stag* as resulting from strategic cooperation, our findings are in contrast with previous meta-analytic results where deliberation was found to undermine pure cooperation but not strategic cooperation^6^. This inconsistency might be accommodated if we consider that, in the mentioned meta-analysis, strategic cooperation is measured in a repeated/sequential setting (where a future opponent’s behavior can be conditional on current actions), whereas, in our experiments, a one-shot simultaneous setting with perfect stranger matching protocol (where no conditional behavior is possible) is used. Indeed, in repeated/sequential settings the role of general trust and that of risk considerations in decision-making is more limited because individuals can observe past actual behavior, and a higher degree of sophistication in reasoning may be triggered by the possibility of conditional behavior.

On an experimental ground, future research should investigate directly what kind of behavior is encoded in the social heuristics for stag hunt interactions. This is likely to require the development of an original research strategy: a direct measurement of what is the believed ruling social norm^60,61^ may be problematic in our context, because the daily-life social norm might be different from the believed ruling norm in the laboratory. On a theoretical ground, since our results suggest that the tension between intuition and deliberation, by conditioning the reliance on social heuristics, influences the decision on which social norm is followed in actual choices, our analysis stimulates the development of a model to study how the prominence of different modes of reasoning co-evolves with the ruling social norm in a given society^46,62,63^.

## Supplementary information

Supplementary Information

## Data Availability

The datasets generated and analyzed during the current study are available from the corresponding author on request.

## Footnotes

1 ^a^The test is implemented with the *ttest* command in *Stata*. Data and routines for replication are available upon request.

2 ^b^Regressions are implemented with the econometric software *Stata*. Data and routines for replication are available upon request.
