# Supplementary material for: Intuition and Deliberation in the Stag Hunt Game
Source: Sci Rep. 2019 Oct 16;9:14833. doi: 10.1038/s41598-019-50556-8 (PMC6795884; doi:10.1038/s41598-019-50556-8)
Supplement: Supplementary file 1 — Supplementary Information [file 41598_2019_50556_MOESM1_ESM.pdf]

Supplementary Information
Appendix for the paper: "Intuition and Deliberation in the Stag Hunt Game"

Marianna Belloc* Ennio Bilancini† Leonardo Boncinelli‡ Simone D'Alessandro§

April 24, 2019

A Instructions (English translation)

Slide 1 - Common to both treatments. Welcome. Thank you for choosing to participate in this experiment.

In this experiment, you will be asked to make choices and you will have the opportunity to earn a certain amount of money. You will be paid privately at the end of the experimental session. It is important that you remain silent for the duration of the experimental session and that you do not try to look at other people's choices in the lab.

If you have any questions, please, raise your hand and one of the experimenters will answer your question. You will be asked to leave the lab if you are found talking in a loud voice, laughing or making gestures, and you will not be paid. We greatly appreciate your cooperation during the experimental session. Please do not leave your sit until you are told.

The experiment consists in participating in some games, each of which requires making a choice on the screen, by using the mouse on your right. Now, please, use your mouse to

*Sapienza University of Rome. Email: marianna.belloc@uniroma1.it.

†IMT School of Advanced Studies, Piazza S.Francesco 19, 55100 Lucca, Italia. Tel.: +39 0583 43 26 737, email: ennio.bilancini@imtlucca.it.

‡University of Florence. Email: leonardo.boncinelli@unifi.it.

§University of Pisa. Email: simone.dalessandro@unipi.it.

click on the “Proceed” button and continue. During the game, you can always consult the instructions sheet.

Slide 2 - Common to both treatments. Before you play each game you will be randomly and anonymously paired with another student of this lab section. The amount of money you will earn depends on your choice and on the choice of your partner. Your partner will not know your identity, just as you will not know his/hers. The interaction will only occur through the computer. You and your partner will simultaneously play the game.

The matching mechanism will be such that you will play each new game with a new partner. In other words, you will never play twice with the same person. None of the people in this lab will know your choices. We ask you not to communicate your choice to anyone of the other participants until the end of the experimental session.

Click “Proceed” to continue. During the game, you can always consult the instructions sheet.

Slide 3 (A) - Control treatment only. The experimental session is organized as follows:

(1) You will play some games.

(2) You will answer to a simple test to verify your understanding of the games.

(3) You will answer a series of questionnaires that will take you about 20 minutes.

(4) You will be told your total score.

(5) You will be invited to leave the room and you will be paid a number of euro equal to your total score.

In the next slide, we will show you the screen that you will be facing during the game and we will explain in detail how to make your choices. We remind you to remain silent and raise your hand if you have any questions.

Follow the instructions on the screen now. We will read the description of what will be shown to you.

Click “Proceed” to continue. During the game, you can always consult the instruction sheet.

Slide 3 (B) - Time pressure treatment only. The experimental session is organized as follows:

(1) Your choice will have to be made within a given time interval that we will communicate later. During the experiment, the time passing will be shown on your screen.

(2) You will play some games.

(3) You will answer to a simple test to verify your understanding of the games.

(4) You will answer a series of questionnaires that will take you about 20 minutes.

(5) You will be told your total score.

(6) You will be invited to leave the room and you will be paid a number of euro equal to your total score.

In the next slide, we will show you the screen that you will be facing during the game and we will explain in detail how to make your choices. We remind you to remain silent and raise your hand if you have any questions.

Follow the instructions on the screen now. We will read the description of what will be shown to you.

Click "Proceed" to continue. During the game, you can always consult the instruction sheet.

Description read by the experimenter for slide 4 - Common to both treatments. On the screen you will see a table that shows the scores you can get playing the game and that will determine the amount of money you can earn at the end. The payoff table that will be shown to you during the games is very similar to the one displayed now.

The same table will be shown to your partner. As we have already said, your score, and how much money you will earn, will depend on the choices done; you will be notified of it at the end of the games.

In this table the payoffs are symbolically represented by X, Y, Z, W (during the game they will be represented by numbers): - if your choice will be A and that of your partner will be A, you will receive $X1$ euros and your partner $X2$ euro; - if your choice will be A and that of your partner will be B, you will receive $Y1$ euros and your partner $Y2$ euro; -

if your choice will be B and that of your partner will be A, you will receive $Z1$ euros and your partner $Z2$ euro; - if your choice will be B and that of your partner will be B, you will receive $W1$ euros and your partner $W2$ euro.

To make your choice you need to click on one of the two buttons below the table (A or B). Once you have made your choice, this cannot be changed.

Click on "Proceed" to proceed. During the game, you can always consult the instructions sheet.

Slide 5 - Common to both treatments. The game will begin shortly. This is your last chance to ask for clarifications. Please, raise your hand if you have any questions for the experimenters. Otherwise, please remain silent and do not communicate with others in any way. Do not leave your sit until you are told.

Slide 6 - Time pressure treatment only. We remind you that you have to make a choice within 10 seconds. You will see time passing during the experiment in the top right corner of the screen.

B Experimental data and analysis

B.1 Summary information on the experiment

Table B.1 reports participation in and structure of the eight sessions.

B.2 Sample statistics

Table B.2 reports the summary statistics describing our sample and comparing the control group with the time pressure treatment group. Two-sample t-tests for mean equality reported in the table document that the two sub-samples are balanced under some relevant respects, such as gender, family background, age, education, experience in game theory and applications. This is true a fortiori if we correct for multiple testing.

Table B.1: Sessions' structure

Session	Treatment	Round	Daytime	#Individuals	#Women	#Obs.	Timed out
1	Control	1,2,3,4	morning	22	12	88	-
2	Control	1,3,4,2	morning	26	13	104	-
3	Control	1,4,2,3	afternoon	26	10	104	-
4	Control	1,4,3,2	afternoon	23	11	92	-
Total obs. in control				97	46	388	-
5	Time pressure	1,4,3,2	afternoon	18	7	72	10
6	Time pressure	1,2,3,4	morning	21	13	84	6
7	Time pressure	1,3,4,2	morning	25	9	100	7
8	Time pressure	1,4,2,3	afternoon	24	12	96	6
Total obs. in time pressure				88	41	352	29
Total obs.				185	87	740	29

Note: Observations and participants by treatment, session, daytime, round, and gender. Last column reports the number of individuals who did not manage to make a decision in 10 seconds under the time pressure treatment.

B.3 Further details on the regression analysis

We estimate the following regression model:

$$stag_{it} = \alpha_d + \alpha_s + \alpha_t + \beta \times pressure_{it} + (\gamma \times basin_g + \boldsymbol{\delta} \times \mathbf{controls}_i) + \epsilon_{it}, \qquad (1)$$

where $stag_{it} = 1$ if individual i chose *stag* in round t and $= 0$ otherwise; $pressure_{it} = 1$ if individual i was under the time pressure treatment in round t and $= 0$ otherwise; $basin_g$ is the basin of attraction of *stag* in game g; α_s, α_d, and α_t are, respectively, session, day, and round fixed effects; ϵ_{it} are the residuals. Bold denotes vectors. Table B.3 reports all regressors described below which are, instead, omitted in Table 1 in main text.

The vector $\mathbf{controls}_i$ includes a set of control variables. The first group measures individual objective characteristics. They are: $female_i = 1$ if individual i is female and $= 0$ otherwise; $graduate_i = 1$ if individual i is enrolled in master studies ('magistrale') and $= 0$ if enrolled in undergrad studies ('triennale'); $father\ education_i$ and $mother\ education_i$ are respectively father's and mother's education levels and are $= 1$ if middle school diploma ('licenza media') or lower, $= 2$ if high school diploma ('maturitá'), $= 3$ if graduation ('laurea'); $inexperience_i = 2$ if individual i had no lab experience and did not take game theory courses, $= 1$ if the individual satisfies one of the two conditions, and $= 0$ if none.

Table B.2: Descriptive statistics

Variable	Min	Max	Mean		P−value
			Control	Time press	diff = 0
female	0	1	0.4742	0.4659	0.9105
			(0.0510)	(0.0535)	
mother education	1	3	2.4742	2.4659	0.9228
			(0.0588)	(0.0625)	
father education	1	3	2.4742	2.3636	0.2530
			(0.0623)	(0.0744)	
age	20	26	22.2474	22.6250	0.2868
			(0.2317)	(0.2691)	
graduate	0	1	0.4948	0.5682	0.3209
			(0.0510)	(0.0531)	
no game theory	0	1	0.5670	0.5227	0.5483
			(0.0506)	(0.0536)	
no lab experience	0	1	0.7629	0.7386	0.7050
			(0.0434)	(0.0471)	

Note: *Female*=1 if the individual is a female and =0 otherwise; *mother educ* is the education of her/his mother (= 1 if middle school diploma ('licenza media') or lower, = 2 if high school diploma ('maturitá'), = 3 if graduation ('laurea')); *father educ* is the education of her/his father (as before); *age* is age; *graduate*=1 if the individual is enrolled in master studies ('magistrale') and =0 if in undergrad studies ('triennale'); *no game theory*=1 if the individual has not taken game theory courses and =0 otherwise; *no lab experience*=1 if she/he has not taken part in previous lab experiments and =0 otherwise. The last column shows the p-value for the test that the corresponding means in the control and time pressure groups are equal (null hp.).

The second group comprises a number of variables capturing participants' responses to a series of questionnaires about relevant subjective characteristics. The first series of questions is aimed at measuring the participant's trust in other people, $trust_i$ and her/his willingness to take risks (in different domains such as in financial investments, sports and leisure, work, health, and social relations), $risklove_i$. Both these indexes vary from 0 (respectively, non trustful and risk averse) to 7 (trustful and risk lover).

The second questionnaire is the Cognitive Reflection Test (CRT) and assesses a person's tendency to override an initial "gut" response to use further reflection and to look for the proper answer to a question or situation to face. We used the variable CRT_i which is equal

Table B.3: Regression analysis - All coefficients

Variable	(1)	(2)	(3)	(4)
pressure	0.1053	0.0996	0.1301	0.1289
	(0.0370)	(0.0364)	(0.0369)	(0.0365)
	[0.0525]	[0.0505]	[0.0507]	[0.0505]
basin				1.0764
				(0.2014)
				[0.1669]
inexperience			0.0275	0.0274
			(0.0293)	(0.0290)
			[0.0406]	[0.0406]
CRT			-0.0152	-0.0142
			(0.0175)	(0.0172)
			[0.0228]	[0.0228]
experiential attitude			-0.0020	-0.0003
			(0.0361)	(0.0354)
			[0.0474]	[0.0473]
rational attitude			-0.0449	-0.0418
			(0.0384)	(0.0374)
			[0.0507]	[0.0504]
risk love			0.0976	0.0950
			(0.0379)	(0.0374)
			[0.0522]	[0.0520]
trust			0.0110	0.0130
			(0.0286)	(0.0283)
			[0.0383]	[0.0383]
female			0.0064	0.0070
			(0.0391)	(0.0383)
			[0.0524]	[0.0524]
graduate			-0.0363	-0.0351
			(0.0398)	(0.0391)
			[0.0539]	[0.0537]
father education			0.0319	0.0315
			(0.0344)	(0.0340)
			[0.0491]	[0.0491]
mother education			0.0549	0.0547
			(0.0378)	(0.0368)
			[0.0483]	[0.0482]
R-squared	0.011	0.067	0.106	0.140
Day Fe	No	Yes	Yes	Yes
Session Fe	No	Yes	Yes	Yes
Round Fe	No	Yes	Yes	Yes

Note: Estimation by OLS. The number of observations is 711. The dependent variable is $stag_{it}$=1 if individual i chose *stag* in round t and =0 otherwise. Standard errors in round brackets are heteroskedasticity robust, while those in square brackets are clustered at the individual level.

to the number of correct answers out of the three proposed.

The third set of questions is the Rational Experiential Inventory (REI-40) which offers the following variables: $rational\ attitude_i$ is the means between a measure the perceived ability to use logical and analytic thinking ("I have a logical mind") and a measure of the perceived reliance on and enjoyment of using logical and analytic thinking ("I enjoy intellectual challenges"); $experiential\ attitude_i$ is the mean between a measure of the perceived ability with respect to one's intuitive impressions and feelings ("I believe in trusting my hunches") and a measure of the perceived reliance on and enjoyment of using feelings and intuitions ("I tend to use my heart as a guide for my actions"). Each of these variables goes from 1 to 5.

B.4 Missing observations due to non-compliance with the time constraint

Observations for which individuals did not make a decision within time lapse imposed by the treatment are somehow censored. So, as a robustness check, we also verify that our main results are not driven by their exclusion from the estimation sample. Since we could not register these 29 potential responses, we run the following robustness check: in a first stage, we predict the censored responses by a probit model that includes all the relevant variables collected for our individuals and then, in a second stage, we estimate our main regressions by using as dependent variable our actual response variable where the 29 missing observations are filled by the values predicted in the first stage. As one would notice, results, shown in Table B.4 and Table B.5, are in line with those reported in Table 1 in the main text.

Table B.4: Second stage with predicted missing observations

Variable	(1)	(2)	(3)
pressure	0.1359	0.1261	0.1606
	(0.0358)	(0.0368)	(0.0389)
	[0.0503]	[0.0511]	[0.0534]
basin			1.1133
			(0.2195)
			[0.1874]
risk love			0.1121
			(0.0404)
			[0.0544]
Day Fe	No	Yes	Yes
Session Fe	No	Yes	Yes
Round Fe	No	Yes	Yes
Individual controls	No	No	Yes
Pseudo R-squared	0.0141	0.0558	0.111

Note: Estimation by probit (marginal effects reported). The number of observations is 740. The dependent variable is $stag_{it}$=1 if individual i chose *stag* in round t and =0 otherwise. Individual controls include: gender, graduate education, father education, mother education, CRT scores, REI40 measures, inexperience in game theory and lab experiments, measure of trust in others, measure of risk love. Standard errors in round brackets are heteroskedasticity robust, while those in square brackets are clustered at the individual level.

Table B.5: Second stage with predicted missing observations

	(1)	(2)	(3)
pressure	0.1057	0.1002	0.1315
	(0.0355)	(0.0347)	(0.0347)
	[0.0504]	[0.0482]	[0.0482]
basin			1.0900
			(0.1943)
			[0.1616]
risk love			0.0946
			(0.0354)
			[0.0490]
R-squared	0.012	0.069	0.144
Day Fe	No	Yes	Yes
Session Fe	No	Yes	Yes
Round Fe	No	Yes	Yes
Individual controls	No	No	Yes

Note: Estimation by OLS. The number of observations is 740. The dependent variable is $stag_{it}$=1 if individual i chose *stag* in round t and =0 otherwise. Individual controls include: gender, graduate education, father education, mother education, CRT scores, REI40 measures, inexperience in game theory and lab experiments, measure of trust in others, measure of risk love. Standard errors in round brackets are heteroskedasticity robust, while those in square brackets are clustered at the individual level.

C Original lab slides

C.1 Instructions

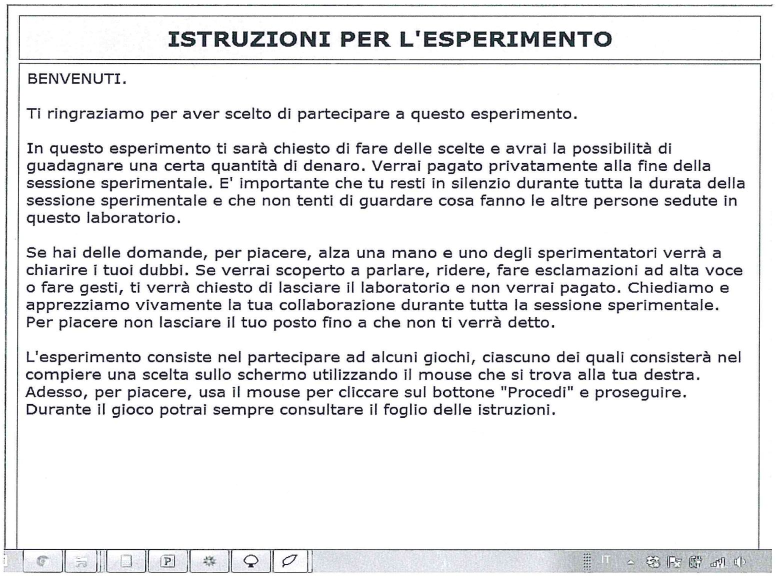

Slide 1 - Common to both treatments.

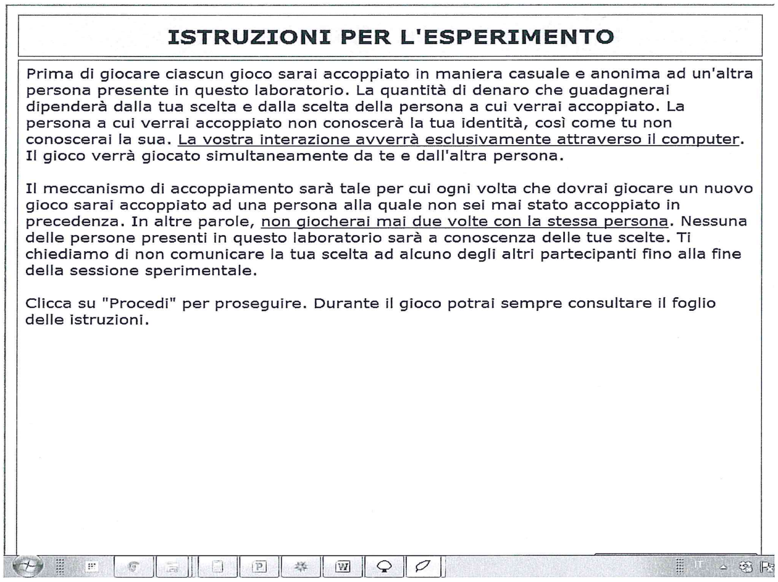

Slide 2 - Common to both treatments.

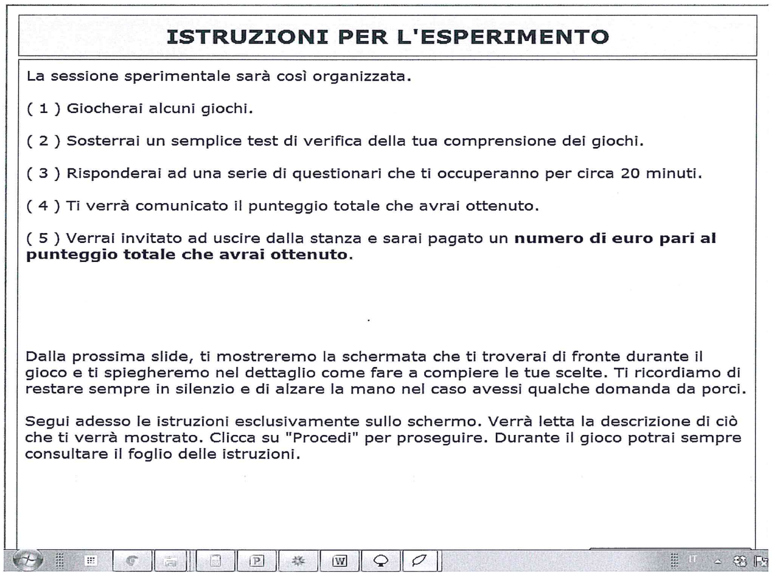

Slide 3 (A) - Control treatment only.

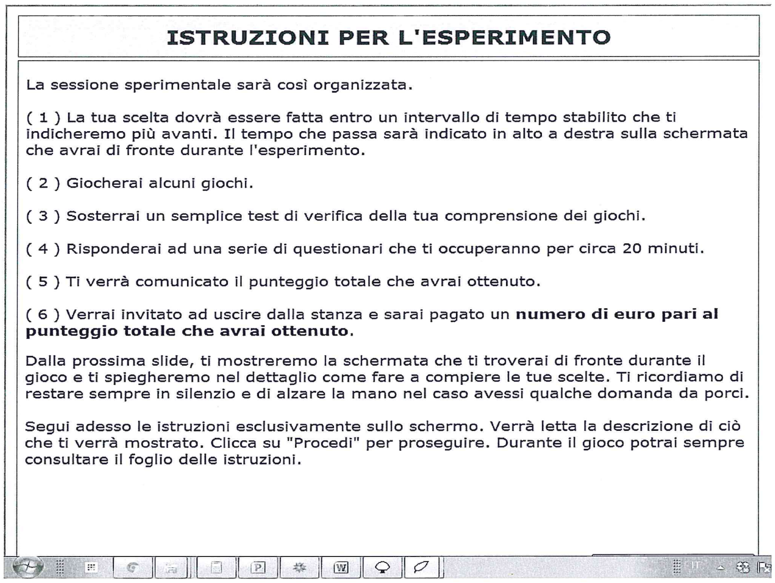

Slide 3 (B) - Time pressure treatment only.

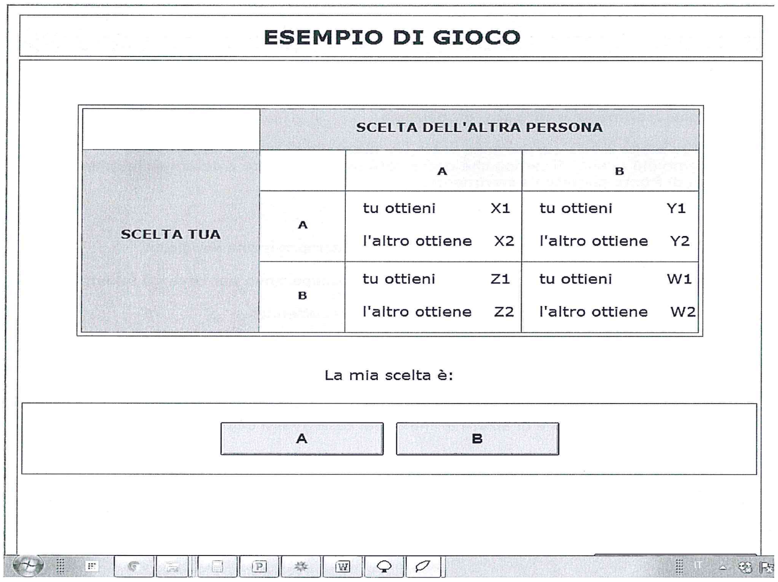

Slide 4 - Common to both treatments.

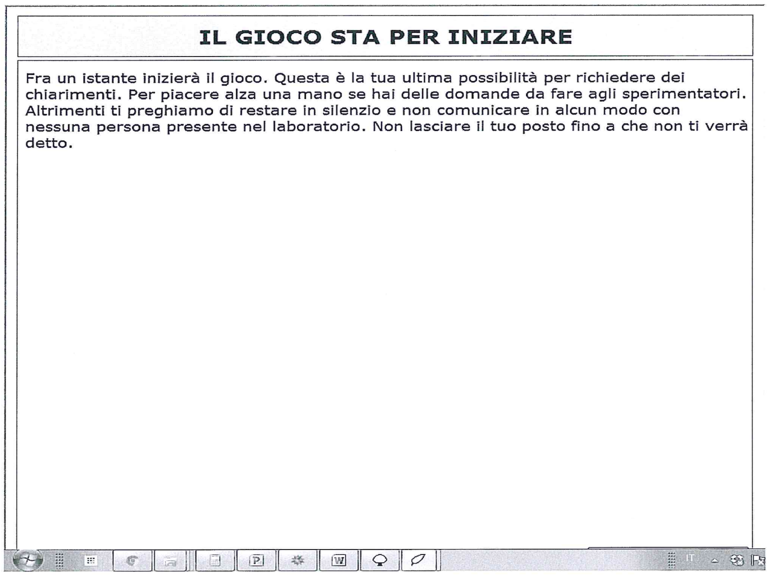

Slide 5 - Common to both treatments.

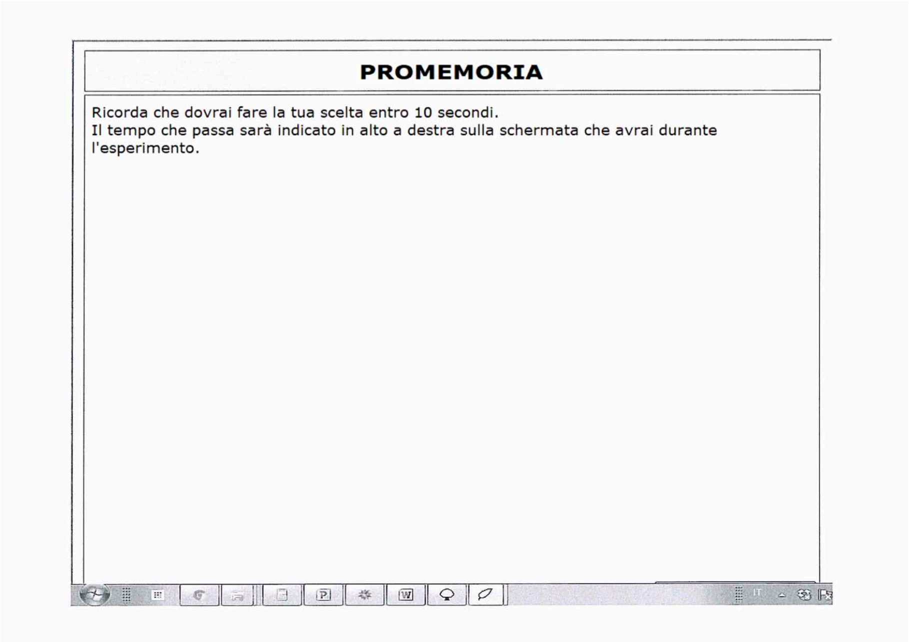

Slide 6 - Time pressure treatment only.

C.2 Risk love and trust

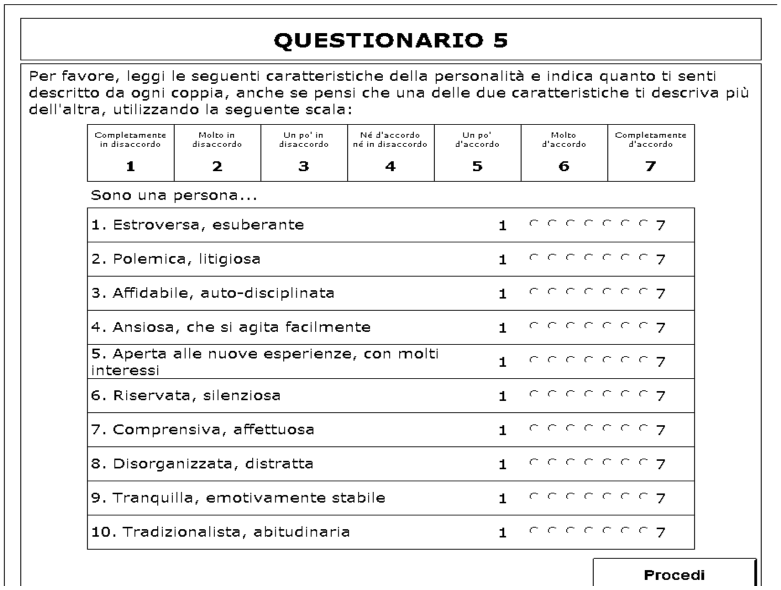

QUESTIONARIO 5

Per favore, leggi le seguenti caratteristiche della personalità e indica quanto ti senti descritto da ogni coppia, anche se pensi che una delle due caratteristiche ti descriva più dell'altra, utilizzando la seguente scala:

Completamente in disaccordo	Molto in disaccordo	Un po' in disaccordo	Né d'accordo né in disaccordo	Un po' d'accordo	Molto d'accordo	Completamente d'accordo
1	2	3	4	5	6	7

Sono una persona...

1. Estroversa, esuberante	1 ○ ○ ○ ○ ○ ○ ○ 7
2. Polemica, litigiosa	1 ○ ○ ○ ○ ○ ○ ○ 7
3. Affidabile, auto-disciplinata	1 ○ ○ ○ ○ ○ ○ ○ 7
4. Ansiosa, che si agita facilmente	1 ○ ○ ○ ○ ○ ○ ○ 7
5. Aperta alle nuove esperienze, con molti interessi	1 ○ ○ ○ ○ ○ ○ ○ 7
6. Riservata, silenziosa	1 ○ ○ ○ ○ ○ ○ ○ 7
7. Comprensiva, affettuosa	1 ○ ○ ○ ○ ○ ○ ○ 7
8. Disorganizzata, distratta	1 ○ ○ ○ ○ ○ ○ ○ 7
9. Tranquilla, emotivamente stabile	1 ○ ○ ○ ○ ○ ○ ○ 7
10. Tradizionalista, abitudinaria	1 ○ ○ ○ ○ ○ ○ ○ 7

Procedi

TIPI Big Five.

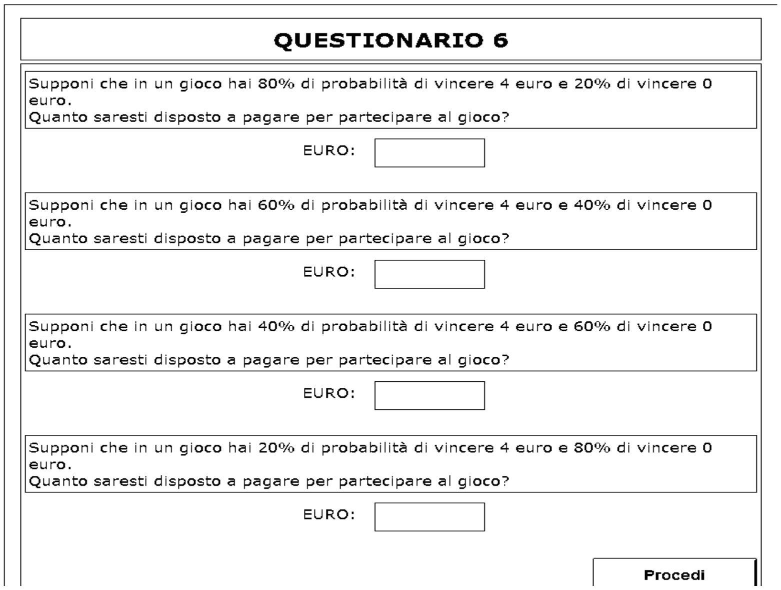

QUESTIONARIO 6

Supponi che in un gioco hai 80% di probabilità di vincere 4 euro e 20% di vincere 0 euro.
Quanto saresti disposto a pagare per partecipare al gioco?

EURO:

Supponi che in un gioco hai 60% di probabilità di vincere 4 euro e 40% di vincere 0 euro.
Quanto saresti disposto a pagare per partecipare al gioco?

EURO:

Supponi che in un gioco hai 40% di probabilità di vincere 4 euro e 60% di vincere 0 euro.
Quanto saresti disposto a pagare per partecipare al gioco?

EURO:

Supponi che in un gioco hai 20% di probabilità di vincere 4 euro e 80% di vincere 0 euro.
Quanto saresti disposto a pagare per partecipare al gioco?

EURO:

Procedi

Risk love.

QUESTIONARIO 7

Per favore, indica quanto sei d'accordo con le affermazioni qui sotto, utilizzando la seguente scala:

Completamente in disaccordo	Molto in disaccordo	Un po' in disaccordo	Né d'accordo né in disaccordo	Un po' d'accordo	Molto d'accordo	Completamente d'accordo
**1**	**2**	**3**	**4**	**5**	**6**	**7**

1. Penso che ci si possa fidare, in generale, delle altre persone	1 ○ ○ ○ ○ ○ ○ ○ 7
2. Penso che le persone siano, in generale, disponibili ad aiutare gli altri	1 ○ ○ ○ ○ ○ ○ ○ 7
3. Penso che le persone abbiano una tendenza ad approfittarsi degli altri	1 ○ ○ ○ ○ ○ ○ ○ 7

Procedi

Trust.
